# Acupuncture Attenuates Anxiety-Like Behavior by Normalizing Amygdaloid Catecholamines during Ethanol Withdrawal in Rats

**DOI:** 10.1093/ecam/neq045

**Published:** 2011-02-14

**Authors:** Zheng Lin Zhao, Guang Wen Zhao, Hou Zhong Li, Xu Dong Yang, Yi Yan Wu, Feng Lin, Li Xin Guan, Feng Guo Zhai, Jia Qi Liu, Chae Ha Yang, Sang Chan Kim, Kee Won Kim, Rong Jie Zhao

**Affiliations:** ^1^Department of Pharmacology, Mudanjiang Medical University, Mudanjiang 157011, China; ^2^Department of Surgery, Medical College of Yanbian University, Yanji 133000, China; ^3^The Research Center for Biomedical Resource of Oriental Medicine, Daegu Haany University, Daegu 706-828, Republic of Korea; ^4^Department of Pharmacology, Medical School of Chonbuk National University, Chonju, Chonbuk 561-180, Republic of Korea

## Abstract

Previously, we demonstrated acupuncture at acupoint HT7 (Shen-Men) attenuated ethanol withdrawal syndrome by normalizing the dopamine release in nucleus accumbens shell. In the present study, we investigated the effect of acupuncture on anxiety-like behavior in rats and its relevant mechanism by studying neuro-endocrine parameters during ethanol withdrawal. Rats were treated with 3 g kg^−1^day^−1^ of ethanol (20%, w/v) or saline by intraperitoneal injections for 28 days. The rats undergoing ethanol withdrawal exhibited anxiety-like behavior 72 h after the last dose of ethanol characterized by the decrease of time spent in the open arms of the elevated plus maze compared with the saline-treated rats (*P <* .05). Radioimmunoassay exhibited there were notably increased concentrations of plasma corticosterone in ethanol-withdrawn rats compared with saline-treated rats (*P* < .05). Additionally, high performance liquid chromatography analysis also showed the levels of norepinephrine and 3-methoxy-4-hydroxy-phenylglycol were markedly increased while the levels of dopamine and 3,4-dihydroxyphenylacetic acid were significantly decreased in the central nucleus of the amygdala of ethanol-withdrawn rats compared with saline-treated rats (*P* < .01). Acupuncture groups were treated with acupuncture at acupoint HT7 or PC6 (Nei-Guan). Acupuncture at HT7 but not PC6 greatly attenuated the anxiety-like behavior during ethanol withdrawal as evidenced by significant increases in the percentage of time spent in open arms (*P* < .05). In the meantime, acupuncture at HT7 also markedly inhibited the alterations of neuro-endocrine parameters induced by ethanol withdrawal (*P* < .05). These results suggest that acupuncture may attenuate anxiety-like behavior during ethanol withdrawal through regulation of neuro-endocrine system.

## 1. Introduction

The most commonly reported reason for relapsing to ethanol consumption after extended periods of abstinence is the desire to relieve the negative emotional responses coming from withdrawal symptoms, such as anxiety, hyperirritability, insomnia and depression [1]. It has been argued that increased anxiety may be the most important negative motivation to relapse to ethanol use among such discomfort components of the ethanol withdrawal [2]. Ethanol withdrawal results in anxiety-like behavior in various kinds of tests of anxiety animals, and it is crucially important to determine whether certain therapeutic manipulations have anxiolytic effect via laboratory tests with anxiety animal models [3–6].

Corticotropin-releasing hormone (CRH) is the major hormonal factor that mediates physiological and behavioral response to external and internal stressors. The dysregulation of brain CRH systems has been implicated in mediating increased anxiety-like behaviors during abused substances withdrawal [7–9]. CRH is secreted by CRH-containing neurons distributed in different brain regions including hypothalamus, nucleus accumbens shell, the bed nucleus of the stria terminalis and central nucleus of the amygdala (CEA) [10, 11]. Among these brain regions, the CEA appears to be particularly important in mediating anxiety-like behavior induced by addicted drugs withdrawal. Rassnick et al. [12] demonstrated that microinjection of a CRH antagonist into the CEA reversed anxiety-like behavior induced by ethanol withdrawal, and Valdez et al. [13] also demonstrated microinjection of a CRH antagonist into the CEA significantly attenuated increased anxiety-like behavioral response to restraint stress during ethanol withdrawal. Chae et al. [14] reported there were significantly increased anxiety-like behaviors in the elevated plus maze (EPM) test and CRH mRNA levels in rat amygdala during nicotine withdrawal. After 2 weeks of withdrawal from repeated intermittent exposure to cocaine, there was great enhancement of CRH-induced long-term potentiation at rat amygdala glutamatergic synapse [15].

Many studies have demonstrated interaction between neurotransmitters and CRH system plays a major role in guiding emotional response to stress [16, 17]. It is well documented that the amygdala was heavily innervated by several neurotransmitter systems including glutamatergic, GABAergic, noradrenergic and dopaminergic systems [18]. Abused drugs such as cocaine, nicotine including ethanol cause alterations of noradrenergic and dopaminergic neurotransmission in the amygdala [19–21], and the altered neurotransmissions produce functional changes of CRH system in the amygdala including increased secretion of CRH [22, 23]. Therefore, it is reasonable to hypothesize that ethanol withdrawal may lead to alterations of norepinephrine (NE) and dopamine (DA) release in the CEA to trigger secretion of CRH further to stimulate secretion of glucocorticoids including corticosterone (CORT) finally to induce anxiety-like behavior in rats.

In traditional Chinese medicine (TCM), health is viewed as the maintenance of balance and harmony between Yin and Yang, while illness is an expression of unbalance and disharmony between Yin and Yang [24]. The balance of Yin and Yang is maintained through smooth flow of “Qi", a metaphysical concept referred to as a vital force or energy in TCM that circulates between the organs along hypothesized channels called “meridians". On these meridians, there are 365 designated acupuncture points (acupoints) that can be used for stimulation through needles or “moxibustion" to balance and harmonize Yin and Yang by improving flow of Qi along the meridians. Based on this notion of TCM, withdrawal from abused drugs can be considered as an imbalance of Yin and Yan in body induced by malfunction of Qi energy system [24]. Therefore, it is conceivable that acupuncture at certain acupoints during withdrawal can correct the disorders of Qi flow through meridians to get body to return to balance and harmony of Yin and Yang. Indeed, several lines of studies showed the efficacy of acupuncture in treating abused drugs withdrawal symptoms [14, 25, 26].

In the previous study, we demonstrated acupuncture at specific acupoint HT7 (Shen-Men) attenuated ethanol withdrawal syndrome by normalizing the DA release in the mesolimbic system [26]. In the present study, we investigated the effect of acupuncture on anxiety-like behavior during ethanol withdrawal and its relevant mechanism in rats.

## 2. Methods

### 2.1. Reagents

Sodium octanesulfonic acid acetonitrile, tetrahydrofurane, NE and 3-methoxy-4-hydroxy-phenylglycol (MHPG), DA and 3, 4-dihydroxyphenylacetic acid (DOPAC) were purchased from Sigma Co. (St Louis, MO, USA). All other drugs were of analytical or high performance liquid chromatography (HPLC) grade.

### 2.2. Apparatus

The EPM (Shanghai Yishu Co., Shanghai, China) consists of a plus-shaped maze that was elevated 50 cm above the ground equipped with a video tracking system. The four arms were each 40 cm long and 10 cm wide. Two opposing arms were enclosed by black wood walls 30 cm high (closed arms); whereas the other two arms were devoid of walls (open arms). The EPM test is based on a natural fear of open and elevated spaces in rodents, the number of entries into open arms and the time spent in open arms are negatively correlated with the anxiety level of the subject.

### 2.3. Animals and Experimental Design

Adult male Sprague-Dawley rats (250–270 g) were obtained from the Laboratory Animal Center in Medical College of Yanbian University (Yanji, China). The rats were individually housed (four rats per cage) under a controlled environment during all experimental treatments. Food and water were provided *ad libitum* and the rats were maintained on a 12-h light/dark cycle. All animal procedures were approved by the Institutional Animal Care and Use Committee and were accomplished in accordance with the provisions of the NIH “Guide for the Care and Use of Laboratory Animals."

The rats were treated with 3 g kg^−1^day^−1^ of ethanol (20%, w/v) or saline by intraperitoneal injections for 28 days. After the last dose of ethanol, rats underwent ethanol withdrawal for 72 h. The acupuncture groups were subjected to acupuncture at acupoint HT7 or PC6 (Nei-Guan) for 1 min once daily for 3 days.

For acupuncture stimulation, stainless steel needles (0.2 mm in diameter) were inserted into the left and right side of the selected acupuncture points. The process of acupuncture stimulation was divided into two parts called reinforcement and reduction. In the reinforcement part, needles were twisted (>360°) thrice a second for 30 s with more strength; in the reduction part, needles were twisted (<180°) once a second with less strength for another 30 s. Two groups of rats were treated with ethanol (ethanol-treated control rats) or saline (saline-treated control rats), respectively, without insertion of acupuncture needles but to be held for 1 min (sham treatment) to get the same restraint as in acupuncture-treated rats.

All rats were tested individually on the EPM for measurement of anxiogenic response just after acupuncture (or sham) treatment. Without any pretest handling, each rat was placed in the center of the maze, after which the cumulative time spent in each arm and the numbers of entries into the open or closed arms were recorded during a 5-min test session. The area inside the center portion (10 × 10 cm) was not considered. Entry by an animal into an arm was defined as beginning when the animal had placed all four paws in that arm. The maze was cleaned with water after each rat had been tested. Exploration of the open arms was encouraged by testing under indirect dim light (2 × 60 W). The behavior in the maze was recorded by a video tracking system. The data recorded as time spent in open arms are expressed as a percentage of total time spent in the arms.

### 2.4. Plasma CORT Assay

After the test on the EPM, rats were immediately killed and decapitated, 1.5 ml of blood was collected into a chilly tube containing EDTA (20 mg ml^−1^, 20 *μ*l) and centrifuged (1000 g) at 4°C for 10 min. The plasma was separated and stored at –80°C until assayed. CORT was measured on plasma samples using the ImmuChem double antibody ^125^I radioimmunoassay (RIA) kit obtained from MP Biomedicals (Orangeburg, NY, USA) and expressed as nanograms per milliliter [27].

### 2.5. Monoamines Analysis

**Figure 1:**
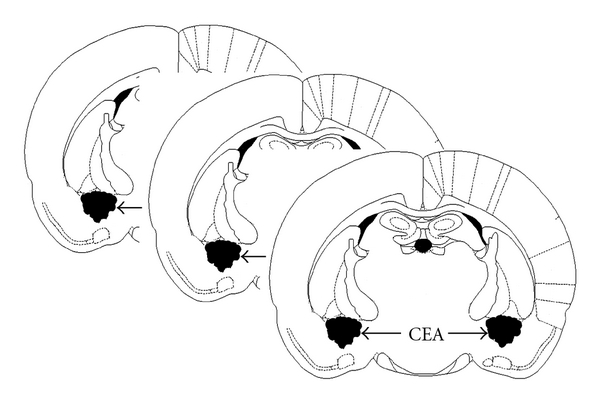
Diagrammatic representation of coronal sections showing the CEA.

To determine concentrations of NE, MHPG, DA and DOPAC in the CEA, eight rats from each group were decapitated, and the entire brain was removed and stored at −80°C. The CEA tissues were punched out according to the protocol established by Wang et al. [28], and the coordinates of CEA were based on the Paxinos and Watson rat brain atlas [29] (Figure 1).

The CEA samples were sonicated in 1 ml of 0.1 M HClO_4_ for 30 s, and centrifuged for 15 min at 26 000 g, 4°C. Then, a 20-*μ*l supernatant aliquot was injected directly into the HPLC with a coulmoetric detector (Coulochem II; ESA, Bedford, MA, USA). The HPLC system consisted of a C18 reverse-phase column (5 U ODS; Altex, Ann Arbor, MI, USA) and an electrochemical transducer with a glassy carbon electrode set at 350 mV. The mobile phase was 0.163 M citric acid, pH 3.0, containing 0.02 mM EDTA with 0.69 mM sodium octanesulfonic acid as an ion-pairing reagent, 4% (v/v) acetonitrile and 1.7% (v/v) tetrahydrofurane. Peaks and values of NE, MHPG, DA and DOPAC in samples were identified and calculated by comparing their retention times and peak heights with those of standards. Results were reported as nanograms per gram protein. The protein concentration in brain homogenate was determined using BCA protein assay.

### 2.6. Statistical Analysis

All data were expressed as mean ± SEM, and analyzed statistically by one-way ANOVA followed by Newman-Keuls multiple comparison tests using the commercially available software GraphPad Prizm 4.0 (GraphPad Software, San Diego, CA, USA). *P* < .05 was considered statistically significant.

## 3. Results

### 3.1. Attenuation of Expression of Anxiety-Like Behavior by Acupuncture

**Figure 2:**
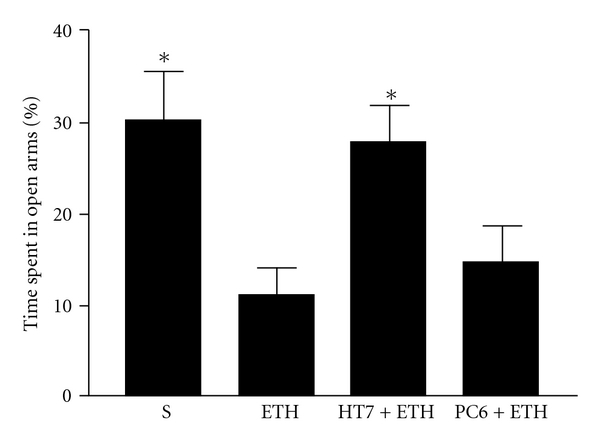
EPM performance. S, saline; ETH, ethanol; HT7, acupuncture at acupoint HT7; PC6, acupuncture at acupoint PC6. Data are expressed as mean ± SEM (*n* = 8) of the percentage of time spent in the open arms of the EPM for a 5-min test period (eight rats per group). *P < .05, compared with ETH group (ANOVA, followed by the post hoc Newman-Keuls multiple comparison test).

The principle of the EPM test is based on a natural fear of open and elevated spaces in rodents, the time spent on open arms is negatively correlated with the anxiety level of the subject. The behavioral data from the EPM test showed the rats undergoing ethanol withdrawal spent less time in open arms (11.12 ± 2.81%, *n* = 8) when compared with saline-treated control rats (30.31 ± 5.13%, *n* = 8, *q* = 4.80, *P* < .05) [*F*(3, 28) = 5.65, *P* < .01], indicating the presence of anxiety-like behavior of ethanol withdrawal. However, rats treated with acupuncture at acupoint HT7 (27.83 ± 3.89%, *n* = 8, *q* = 4.18, *P* < .05) but not PC6 (14.69 ± 3.81%, *n =* 8, *q* = 0.89, *P* > .05) showed significant increases in the percentage of time spent in open arms compared with ethanol-treated control rats (Figure 2). The percentage of time spent in open arms was calculated as follows:

(1) $$\begin{array}{cc} & \text{Percentage}\;\text{of}\;T_{\text{spent}\;\text{in}\;\text{open}\;\text{arms}}\;\\ & \;\;=\frac{T_{\text{spent}\;\text{in}\;\text{open}\;\text{arms}}}{\left(T_{\text{spent}\;\text{in}\;\mathrm{closed}\;\text{arms}}\;+T_{\text{spent}\;\text{in}\;\text{open}\;\text{arms}}\right)}. \end{array}$$

The alteration of locomotor activities is also an important behavioral parameter to show ethanol withdrawal signs in rats. Horizontal locomotor activity also was recorded by video tracking system during 5-min test session. However, there was no significant difference observed in the total ambulatory distance among the different groups (data not shown).

### 3.2. Inhibition of CORT Secretion during Ethanol Withdrawal by Acupuncture

**Figure 3:**
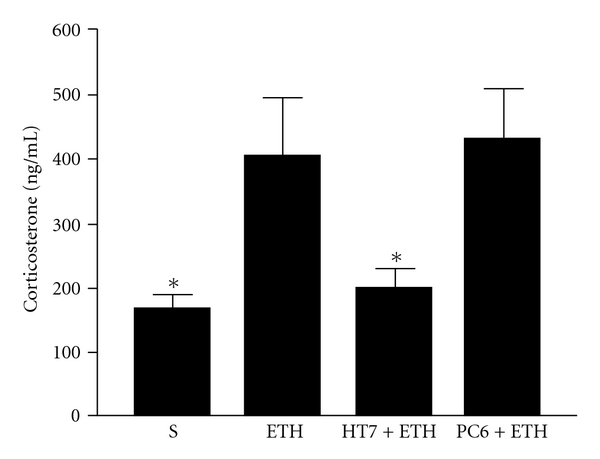
Plasma concentrations of CORT during ethanol withdrawal. S, saline; ETH, ethanol; HT7, acupuncture at acupoint HT7; PC6, acupuncture at acupoint PC6. Data are expressed as mean ± SEM (*n* = 8) of the concentration of plasma CORT. *P < .05, compared with ETH group (ANOVA, followed by the post hoc Newman-Keuls multiple comparison test).

To investigate whether the improvement of anxiety-like behavior by acupuncture treatment was associated with its effect on changes of plasma CORT levels during ethanol withdrawal, the concentrations of plasma CORT were measured by RIA. As measured at 72 h after the last dose of ethanol, the levels of plasma CORT significantly increased in ethanol-treated control rats (406.58 ± 88.61, *n* = 8) compared with saline-treated control rats (169.17 ± 19.45, *n* = 8, *q* = 3.87, *P* < .05) [*F*(3, 28) = 4.96, *P* < .01]. In accordance with the behavioral data, the treatment with acupuncture at HT7 (202.24 ± 29.42, *q* = 3.33, *P* < .05) but not PC6 (433.70 ± 77.26, *q* = 0.44, *P* > .05) produced significant reduction of plasma CORT levels compared with sham treatment during ethanol withdrawal (Figure 3).

### 3.3. Normalization of Levels of NE, MHPG, DA and DOPAC in the CEA during Ethanol Withdrawal by Acupuncture

To examine possible mechanisms of the anxiolytic effect of acupuncture at HT7, the concentrations of NE and its major metabolite MHPG and the concentrations of DA and its major metabolite DOPAC in the CEA were determined during ethanol withdrawal. HPLC analysis showed that there was significant increases in the concentrations of NE and MHPG in the CEA 72 h after the last injection of ethanol in ethanol-treated control rats when compared with saline-treated control rats (*P* < .01), whereas the concentrations of DA and DOPAC in the CEA were markedly decreased during ethanol withdrawal (*P* < .01). Treatment with acupuncture at HT7 (*P* < .05 or .01) but not PC6 (*P* > .05) significantly inhibited the alterations of monoamines levels in the CEA during ethanol withdrawal. These results indicated that the anxiolytic effect of acupuncture at HT may be mediated by regulating monoamine levels in the CEA during ethanol withdrawal (Table 1).

## 4. Discussion

Designed to assess internal conflict between voluntary approach and withdrawal tendencies, the EPM is the most frequently used tool to measure withdrawal induced anxiety-related behavior in both rats and mice [30]. Many studies demonstrated that addicted substances such as nicotine and ethanol exhibited robust anxiety-like behavior in the EPM system during withdrawal period characterized by the reduced number of entries in open arms or the decrease of percentage of time spent in open arms [3, 14]. In our present study, we observed that rats undergoing ethanol withdrawal spent much less time in open arms than saline-treated control rats. These results are consistent with the data from the similar studies done by others [3, 4].

Increased locomotor activities are also regarded as important behavioral changes to show ethanol withdrawal signs in rats. Kim et al. [25] demonstrated there were markedly enhanced locomotor activities in rats at 24, 48 and 72 h after termination of daily injections of ethanol for 21 days. Additionally, in the previous study, we also observed significant increases of locomotor activities in rats following 48 h of ethanol withdrawal [26]. However, in the present study, there was no significant difference recorded in the total distance traveled among the four groups. This discrepancy in locomotor activities of ethanol-withdrawn rats may be potentially due to the different spaces where the locomotor activities were measured, since in the previous study the space used for measurement of locomotor activities was a rectangular container (40 cm × 40 cm × 45 cm), while in the present study the locomotor activities were recorded in the arms of the EPM. On the other hand, no significant difference in locomotor activities among the four groups in the present study indicates that the difference in time spent in the open arms of the EPM did not come from enhanced exploratory activities but from the anxious inner state of rats.

Numerous studies have shown that the test in the EPM is also sensitive to the actions of anxiolytic agents. Widely used classic anxiolytics, diazepam and alprazolam, showed robustly inhibitory effect on rodents anxiety-like behavior in the EPM test [31, 32]. Currently the pharmacological treatment of anxiety mainly relies on the benzodiazepines [33]. However, for well-known reason, there is a great limitation in chronic using of benzodiazepines [34].

Being one of the best known complementary and alternative medical treatments, acupuncture recently becomes more popular in treating mental disorders such as insomnia, anxiety, depression, algesia and drugs addiction due to the great benefits from its effectiveness, quickness, inexpensiveness and safety [35–37]. Lee et al. [38] demonstrated acupuncture at HT7 and PC6 is a useful therapeutic method for post-stroke-onset insomnia as it reduces sympathetic hyperactivities. Wu et al. [39] reported the therapeutic effect of acupuncture at some acupoints including GV20 (Bai-Hui), LI4 (He-Gu), LR3 (Tai-Chong), HT7 and PC6 on post-stroke anxiety symptoms based on clinical observation. Roschke et al. [40] observed in a single-blind, placebo-controlled study design that patients who experienced acupuncture improved major depression symptoms more than patients treated with mianserin alone. Although the beneficial effects of acupuncture on anxiety and depression were reported by many clinical studies, the confirmation of its effectiveness still remains controversial since there were some data from clinical observation exhibited that acupuncture effect on anxiety and depression was not better than the placebo manipulation itself [41].

Acupuncture is a complex intervention because of the difficulty in precisely defining what the active ingredients are and how they relate to each other. Moreover, due to the lack of a consensus method to develop a standardized treatment protocol for randomized controlled trials (RCTs), it is difficult to evaluate the effect of acupuncture on mental disorders in a more qualitative and quantitative way. Therefore, it becomes more important than ever to conduct laboratory experiments based on RCTs to provide experimental database for clinical practice. Park et al. [42] demonstrated acupuncture at HT7 markedly ameliorated anxiety-like behavior in adult rats following maternal separation by modulating the NPY system in amygdala. Lee et al. [36] reported that stimulation on acupoint PC6 suppressed the symptopathology of the hypo-activated hypothalamus-pituitary-adrenaline axis in chronic CORT-induced rat model of depression. In the previous study, we also demonstrated acupuncture at acupoint HT7 attenuated ethanol withdrawal syndrome by normalizing DA release in nucleus accumbens shell [26]. In the present study, there was great inhibition of anxiety-like behavior in the rats that received acupuncture at HT7 during ethanol withdrawal compared with the rats that received acupuncture at PC6 or sham-treatment evidenced by increased percentage of time spent in the open arms in the EPM test. It indicated that acupuncture at specific acupoint HT7 may have preventive effect on anxiety-like behavior induced by ethanol withdrawal in rats.

The therapeutic effect of acupuncture on ethanol withdrawal anxiety was also supported by the data from RIA of plasma CORT showing that acupuncture at HT7 significantly inhibited the enhancement of plasma CORT levels during ethanol withdrawal. CORT is a main glucocorticoid in rodents, involved in regulation of immune reactions and stress responses [43]. As seen in many studies the increased plasma level of CORT is a most important hormonal hallmark of an anxiety state of body, and was reduced by several anti-anxiety manipulations [44].

Secretion of CORT is regulated by CRH-adrenocorticotropic hormone axis and the increased level of CORT reflects the hyperactivity of neuro-endocrine stress system in brain. CRH, a key hormone operating in concert with catecholamines and other neuotransmitters plays a pivotal part in the control of anxiety-like behavior in rodents and humans [43]. Noradrenergic and dopaminergic afferents from brain stem are two major innervations of the CRH-containing regions in amygdala [22, 45, 46], and NE is putatively regarded as the main secretagogue to induce CRH release [47]. Previously, we demonstrated local injection of nicotine into nucleus tractus solitarius (NTS) induced NE release in the amygdala through activation of NMDA glutamate receptor in the NTS [45]. In another study, we also demonstrated there was a significant decrease of DA release in nucleus accumbens shell of rats during ethanol withdrawal [48]. Additionally, the studies done by others also demonstrated that rats chronically exposed to ethanol presented significant alterations in the levels of NE, MHPG, DA and DOPAC in brain [49, 50]. In the present study there were significant increased levels of NE and MHPG and markedly decreased levels of DA and DOPAC in the CEA during ethanol withdrawal.

NE and CRH systems in the extended amygdala are implicated in the anxiety response that occurs during withdrawal from long-term opiates, cocaine, ethanol and cannabinoids [51]. And many of these stress-associated behaviors are reversed by noradrenergic or CRH antagonists given systemically or locally into the extended amygdala [13, 52, 53]. It is well known that dopaminergic projection from VTA to nucleus accumbens plays a central role in mediating positive reinforcement effect of abused drugs. Additionally, some evidences showed that the decrease in function of the mesolimbic dopaminergic circuits may have its part in mediating negative reinforcement effect of abused drugs during withdrawal. Behavioral analysis and microdialysis showed withdrawal of rats from chronic ethanol resulted in withdrawal symptomatology and dramatic fall in extracellular DA in the mesolimbic dopaminergic system [26, 48]. Shen also reported that ethanol withdrawal reduced the number of spontaneously active VTA dopamine neurons in conscious animals [54]. It is well documented that therapeutic effects of acupuncture on central nervous system are mediated by modulating several neurotransmitter systems such as catecholamines, glutamate and GABA [55]. In the present study, acupuncture at HT7 significantly suppressed the elevated levels of NE and MHPG and markedly increased the lowered levels of DA and DOPAC in the CEA during ethanol withdrawal. These results indicated that acupuncture at HT7 may correct the dysregulation of catecholamines in the CEA during ethanol withdrawal to normalize hyper-activated hormonal (CRH, ACTH and CORT) responses further to reduce the expression of anxiety-like behavior in rats.

Destroyed balance between Yin and Yang is regarded as fundamental cause of diseases in TCM, and the maintaining of homeostasis in body functions is an important target for complementary and alternative medical therapy [56]. Acupuncture corrects the imbalances in Yin and Yang to maintain homeostasis of body by facilitating stagnant Qi flow caused by adverse forces including chronic exposure to ethanol through stimulation on certain acupoints along the meridians [25, 48]. It is noteworthy that Qi is referred to as energy in TCM and the balance of energy in body is most critical in rehabilitating the ethanol-dependent body. Reinforcing and reducing methods are needle manipulation methods in acupuncture to treat deficient or excessive syndromes [57]. Withdrawal syndrome is also regarded as coexistence of excessive activation of neuro-endocrine systems, which elicit anxiety response and hypo-activation of neuro-endocrine systems that mediate happy and comfort feelings in body. Based on these notions, in the present study, we also employed reinforcing and reducing manipulation methods in acupuncture treatment.

Classical texts describe most of the main acupoints as existing on the 12 main and 8 extra meridians through which Qi flows, and the main meridians have their corresponding names according to TCM theory of “Zang-Fu" [58]. HT7 is the Yuan Source point of Heart Channel of Hand Shao-Yin, which has been frequently used to treat mental disorders including drug addiction [26, 48, 58]. PC6 is the Luo Connecting point of Pericardium Channel of Hand Jue-Yin, has also been widely used to treat mental disorders such as insomnia and depression [38, 58]. In the present study, acupuncture at acupoint HT7 but not PC6 corrected the abnormalities in behavioral and neuro-endocrine parameters during ethanol withdrawal in rats. It indicates that acupuncture may have therapeutic effect on anxiety induced by ethanol withdrawal, and HT7 is an important acupoint in treating anxiety-like behavior during ethanol withdrawal.

**Figure 4:**
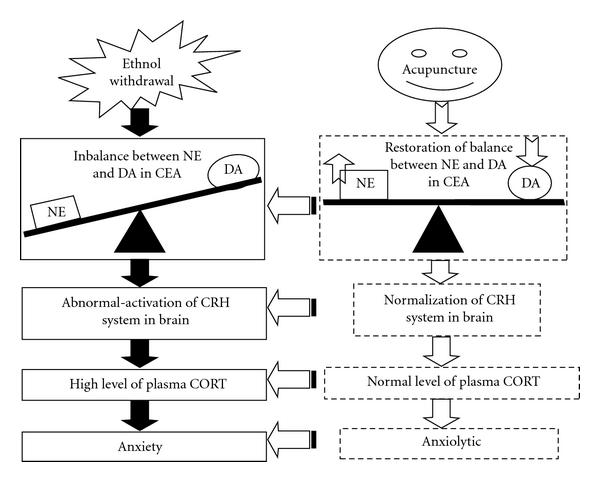
Mechanisms of anxiolytic action of acupuncture.

In summary, in the present study, we observed preventive effect of acupuncture at specific acupoint HT7 on the expression of anxiety-like behavior and the over-secretion of CORT during ethanol withdrawal in rats. Additionally, acupuncture at HT7 also inhibited the increase in the levels of NE and MHPG and the decrease in the levels of DA and DOPAC in CEA induced by ethanol withdrawal. These results suggest that acupuncture can prevent the emergence of an anxiety state in body during ethanol withdrawal by correcting the imbalance of neurotransmitters in brain disturbed by chronic ethanol (Figure 4).

## Funding

Research Fund for Overseas & Returned Scholars from the Heilongjiang Provincial Department of Education of China (No. 1154h22).

## Figures and Tables

**Table 1:** Normalization of the levels of NE, MHPG, DA and DOPAC in CEA during ethanol withdrawal by acupuncture.

Groups	NE	MHPG	DA	DOPAC
S (8)	237.33 ± 18.72**	98.13 ± 10.08**	798.29 ± 67.32**	223.77 ± 19.54**
ETH (8)	735.67 ± 81.83	300.24 ± 34.23	430.18 ± 56.77	109.65 ± 22.29
HT7 + ETH (8)	329.17 ± 30.11**	137.09 ± 12.87**	690.34 ± 102.23*	198.18 ± 25.47*
PC6 + ETH (8)	688.12 ± 100.32	277.12 ± 23.31	399.37 ± 34.65	134.21 ± 23.33
*F* (3, 28); *P*-value	13.99; *<*0.01	20.37; *<*0.01	7.89; *<*0.01	5.51; *<*0.01

Data are presented as mean ± SEM protein (ng g^−1^) in the CEA from rats sacrificed 72 h after the last dose of ethanol or saline. The numbers in parentheses indicate the number of rats in each group. *P < .05, **P < .01, compared with ETH group (ANOVA, followed by the post hoc Newman-Keuls multiple comparison test).

NE, norepinephrine; MHPG, 3-methoxy-4-hydroxy-phenylglycol; DA, dopamine; S, saline; ETH, ethanol; HT, acupuncture at acupoint HT7; PC6, acupuncture at acupoint PC6.
